# Emotional Intelligence and Prefrontal Cortex: a Comparative Study Based on Wisconsin Card Sorting Test (WCST)

**Published:** 2011

**Authors:** Ahmad Alipour, Zahra Arefnasab, Abdolreza Babamahmoodi

**Affiliations:** 1 Payame Noor University (PNU), Tehran, Iran; 2 Department of Research and Technology, Baqiyatallah University of Medical Sciences (BMSU),Tehran, Iran

**Keywords:** Emotional intelligence (EI), Prefrontal cortex (PFC), Wisconsin Card Sorting Test (WCST)

## Abstract

**Objective:** Emotional intelligence (EI) is a set of competencies that enable us to engage in sophisticated information processing of emotions and emotion-relevant stimuli and to use this information as a guide for thinking and behavior. Prefrontal cortexes (PFC) of brain and related regions have an important role in emotion and emotional regulation. Accordingly, we conducted a study to investigate the relation between EI and performance in Wisconsin Card Sorting Test (WCST) (a neuropsychological test, used to evaluate some of the frontal lobe functions).

**Methods:** In this quasi-experimental study, 250 volunteers from BS and BA students of universities of Tehran were recruited using available sampling method. Bar-on EI, general health questionnaire (GHQ-28) and Raven's Progressive Matrices were completed by the participants. They were categorized into two groups; each group contained 40 students with high and low EI, whose performance in WCST were evaluated thereafter individually. Data was analyzed by MANOVA.

**Results:** Our results showed that the high EI group had a better performance in WCST than the low EI group.

**Conclusion:** It can be concluded that people with better EI may have better PFC functions.

## Introduction

When psychologists began to write and think about intelligence, they focused on its cognitive aspects such as memory and problem-solving. However, there were researchers who recognized early that the non-cognitive aspects were also important. Recently, the construct of emotional intelligence (EI) has emerged in the popular literature as an additional explanatory concept for human behavior and performance. EI, the ability of conceptualization, proposed by Mayer, Salovey, and their colleagues, refers to a set of competencies that enable us to engage in sophisticated information processing of emotions and emotion-relevant stimuli and to use this information as a guide for thinking and behavior. It involves the perception, assimilation, comprehension, and management of emotions (1). In fact, EI has two key components: (*i*) Strategic EI is the competency to understand (to realize the causes of emotions) and manage emotions (to figure out effective strategies that apply emotions helping to achieve a goal); and (*ii*) Experiential EI is the competency to perceive (to correctly identify how people are feeling) and use (to integrate feelings into thinking) emotions (2). It has also been described as a form of social intelligence that involves the ability to monitor one’s own and others’ feelings and emotions, to be discriminate among them, and to use this information to guide one’s thinking and action. EI has as much to do with knowing when and how to express emotion as it does with controlling it. It is held to explain how emotions advance life goals. Its proponents consider it to be distinct from either general cognitive ability (g-factor) or personality. While different theories of EI have been proposed, there is still controversy about how EI should be conceptualized and measured. It is agreed, however, that EI’s relevance depends on it being able to predict significant life outcomes (3).

Regarding the neural basis, emotions began to evolve from the olfactory lobe. The limbic system slowly became refined, and the thinking brain (neocortex) evolved. The amygdale, a key factor in developing our EI, is the emotional nerve centre of the brain that responds before the neocortex. The amygdale’s function as a mechanism is often referred to as “emotional hijacking”. When emotional hijacking occurs, the prefrontal cortex (PFC) acts as a manager of emotions by regulating and weighing reactions before acting on them (4).

A network can compute a kind of emotional coherence through interactions among multiple brain areas including the PFC and the amygdale (1). The frontal cortex plays an important role in the interpretation, expression and regulation of emotion. Amygdale has reciprocal connections with medial frontal lobe regions, which suggest that medial frontal cortex is in a position to regulate and organize emotion–based actions. Like medial frontal cortex, orbital frontal cortex has reciprocal connections with the amygdale. This suggests that this area is a site of integration of world representation and emotional processing (5).

Researches explain that PFC is a crucial component for EI, especially according to its connection to the limbic system (2).

Wisconsin Card Sorting Test (WCST) is a famous neuropsychological test that was designed in 1948 by Grant and Berg as an index of abstract reasoning, concept formation, and response strategies to changing contextual contingencies (6). Many years later, Milner introduced the WCST to assess prefrontal lobe dysfunction in patients with brain lesions (7). Nowadays, there are at least two different systems of administration and scoring of the WCST; the standard version by Grant and Berg (1948) with Milner´s (1963) correction criteria and the shortened version by Heaton (8). Several classic studies reported the sensitivity of the WCST to frontal lobe lesions (9). Although there is some debate about the use of WCST only for assessing frontal lobe dysfunction, using it as a valid test continues.

As mentioned before, PFC has an important role in emotion and EI. Thus, this study was performed to assess the differences between performances of high and low EI groups in WCST test (one of the most famous neuropsychological tests) and accordingly, to conclude that this difference is due to differences in PFC function.

## Materials and Methods

*Subjects:*

In this quasi-experimental study, 250 volunteers from BS and BA students of universities of Tehran were recruited using available sampling method. They aged from 20 to 30 years. The Bar-on questionnaire of EI, GHQ-28 and Raven's Progressive Matrices were completed by them in their classrooms. Our exclusion criteria were positive past history of any psychological or neurological problems, head injury and decreased level of consciousness. Examinees performed general health questionnaire (GHQ-28) test and abnormal persons were omitted according to the obtained results. In addition, the participants who had EI scores between “mean score –SD” and “mean score +SD” were omitted based on the results of EI questionnaire. For controlling the effect of IQ, we used BS and BA students with the assumption that they have at least normal IQ, but we omitted the persons with high IQ (more than 110) by using the Raven's Progressive Matrices (five persons). Finally, assuming EI scores, we formed two sample groups, each group with 40 subjects. The group, in which the EI scores were “mean score +SD" was called high EI, whereas the other group with the EI as “mean score –SD” was called low EI.

The goals of our study were explained by experts to the subjects and they had informed cooperation and participation in the tests.

*Bar-on EI questionnaire:*

This questionnaire is a self-report questionnaire and has 90 questions that evaluate 15 factors of EI. These factors include emotional self-awareness, self-esteem, assertiveness, self-actualization, independency, empathy, interpersonal relationship, social responsibility, problem solving capacity, reality testing, flexibility, tolerance to stress, control of impulsion, happiness and optimism. This questionnaire, was first designed in 1980, and it was the first reliable ultra-cultural questionnaire for evaluating EI that was revised in 1997 (10). In Iran, this questionnaire was standardized in 2005 by Dehshiri in students of Tehran University and by Samuie in students of Esfahan University. In Dehshiry’s study, test-retest reliability score was 0.735 and Cronbach’s alpha score was 0.733. Besides, the reliability scores were 0.88 and 0.93 in Samuie’s study (11).

*Wisconsin Card Sorting Test:*

In the Heaton form, WCST consists of four key cards and 128 response cards with geometric figures that vary according to three perceptual dimensions (color, form, or number). The task requires subjects to find the correct classification principle by trial and error and examiner’s feedback. Once the subject chooses the correct rule, they must maintain this sorting principle (or set) across changing stimulus conditions while ignoring the other-now irrelevant-stimulus dimensions. After ten consecutive correct matches, the classification principle changes without warning, demanding a flexible shift in set. The WCST is not timed and sorting continues until all cards are sorted or a maximum of six correct sorting criteria have been reached. Although Heaton’s correction norms offer sixteen different scores, due to the internal structure of the test, many authors normally rely on no more than two or three scores as an index of subject’s performance including number of categories completed, number of preservative errors, and number of nonperseverative errors (12,13). In this study, we also used Heaton’s correction norms and three scores.

*General Health Questionnaire (GHQ-28):*

General Health Questionnaire (GHQ) was introduced by Goldberg and Hillier in 1979. It is simple to be administered, easy to be completed and scored and is widely used in many studies of well-being. Obtained from the assessments of psychological well-being, GHQ can be useful in understanding various sources of distress, as well as any predisposing factors. Multiple linear regressions showed that depression, anxiety, self-esteem and stress were significant independent predictors of GHQ scores (14). Possibly, the most common questionnaire of mental well-being is the GHQ. It developed as a screening tool to detect those likely to have or be at risk of developing psychiatric disorders. It is a measure of the common mental health problems/ domains of depression, anxiety, somatic symptoms and social withdrawal. Available in a variety of versions using 12, 28, 30 or 60 items, the 28-item version is used most widely (15). GHQ test got standardized in Iran previously (16). In a study from Iran, coefficients of criterion validity, structural validity and reliability showed that GHQ-28 is one of the most valid instruments for screening general health (17).

*Raven's Progressive Matrices:*

Raven's Progressive Matrices (also Raven Progressive Matrices) are widely used as non-verbal intelligence tests. The test is supposed to measure the educative ability or the ability to extract and understand information from a complicated situation and it is often used as a test of general intelligence (18). In each test item, one is asked to find the missing pattern in a series. Each set of items gets progressively harder, requiring greater cognitive capacity to encode and analyze. They are offered in three different forms for different ability levels, and for age ranges from five through adult: Colored Progressed Matrices (younger children and mental retard groups), Standard Progressive Matrices (average 6 to 80 years of age), and Advanced Progressive Matrices (above average adolescents & adults). In our study, we used standard form (19).

*Statistics:*

For analyzing the data, multivariate analysis of variance (MANOVA) was used. The MANOVA is a type of multivariate analysis used to analyze data that involves more than one dependent variable at a time. MANOVA allows us to test hypotheses regarding the effect of one or more independent variable(s) on two or more dependent variables. In our study “groups with high or low EI” were independent variables and dependent variables included "number of categories formed”, “number of perseveration errors” and “number of other errors”.

## Results

Descriptive statistics (mean and standard deviation) of EI questionnaire and three aforementioned dependent variables are presented in table 1.

There were differences between mean scores of two groups according to three dependent aforementioned variables. Significance of these differences is discussed in table 2.

**Table 1:** Mean and standard deviation of EI questionnaire and three aforementioned dependent variables

	High EI group		Low EI group	
	*M*	*SD*	*M*	*SD*
Total Score of EI	367.43	3.39	246.23	4.69
Number of Categories Formed	5.30	0.93	3.75	0.067
Number of Perseveration Errors	6.70	4.36	11.97	4.58
Number of Other Errors	12.52	4.95	20.80	8.37

**Table 2:** Differences between high and low EI groups in “number of categories formed”, “number of perseveration errors” and “number of other errors”

*Dependent Variables*	*Mean* *Square*	*Sum of* *Square*	*F*	*DF*	*Sig*
Number of Categories Formed	48.05	48.05	72.21	1	0.001
Number of Preservative Errors	556.51	556.51	227.80	1	0.001
Number of Other Errors	1369.51	1369.51	28.91	1	0.01

Significant differences were observed between two groups in “number of categories formed” (F=72.21, P=0.001), “number of perseveration errors” (F=7.80, P=0.01) and “number of other errors” (F=28.91, P=0.001).

These data suggested that the high EI group had fewer total error and perseveration error than low EI group. In addition, this group (high EI) had more categories formed than the other group (low EI). Overall, high EI group’s function in WCST was better than the other group.

## Discussion

This study was performed to investigate the function of PFC in high and low EI groups. Based on the assumption that PFC plays a crucial role in human social-emotional behavior, we used the WCST in individuals with high and low EI. According to our researches, there was not any comparable published study in data bases.

Our findings showed that performance of individual with high EI in WCST is better than people with low EI that may indicate better PFC function in the high EI group. These findings are parallel to previous studies of the neural basis of EI and role of PFC and related regions of brain in processing the knowledge that is crucial for understanding and managing emotionally relevant information (2). In addition, our finding of poor WCST performance in low EI people complements a previous study that demonstrated an association between ventromedial PFC damage and impaired EI measured by the Emotional Quotient Inventory (20). Ventromedial PFC damage results in social incompetence, decreased sensitivity to social and situational stimuli, interpersonal interaction problem, and abnormal changes in mood and personality (20-23) related to EI. Our results were in accordance with previous studies that have shown that the behavioral and emotional dysfunction associated with ventromedial PFC damage cannot be explained by impaired cognitive intelligence measured by standard intelligence tests (20, 24). It emerged from our findings that those competencies underlying EI have clear neural foundations and can be impaired despite normal intellectual functioning. On the other hand, EI complements cognitive intelligence and permits the individual to evaluate emotional and social processes in decision making (25).

In this study, we highlighted the role of PFC as a key component of EI. However, we know that other regions of brain such as subcortical limbic structures, amygdale (which mediates emotional processes) and closely associated regions such as the insula, cingulate cortex, and parietal cortices influence the emotionally related behaviors as well (20,2). For better understanding of the neural basis of EI, evaluating the function of these regions is necessary. Furthermore, for evaluating PFC function, due to some restrictions, we only used one neuropsychological test (WCST). In this regard, to obtain better and more accurate data, implementing several neuropsychological tests together or using these tests with other more accurate methods like neuroimaging methods can be recommended.

## Authors’ Contribution

AA conceived and designed the study. ZA participated in designing the evaluation, collected the data, interpreted them, performed the statistical analysis and revised the manuscript. AB re-evaluated the data, revised the manuscript and performed the statistical analysis and drafted the manuscript. All authors read and approved the final manuscript.

## Notes

**Declaration of Interest:** None.
